# Transgenerational trauma and attachment

**DOI:** 10.3389/fpsyg.2024.1362561

**Published:** 2024-04-08

**Authors:** Zlatomira Kostova, Vanya L. Matanova

**Affiliations:** ^1^Plovdiv University “Paisii Hilendarski”, Plovdiv, Bulgaria; ^2^Institute of Mental Health and Development (Bulgaria), Sofia, Bulgaria

**Keywords:** attachment trauma, transgenerational transmission, depression, anxiety, case description

## Abstract

An integrative approach is presented to understand the transmission mechanisms of attachment trauma and the quality of the internal working model through manifestations of bodily symptoms concerning physical and mental health. A case of dissociative symptomatology is described in a woman whose ancestors experienced individual and collective trauma related to the political regime. The approach allows for an analytical view beyond the standard complaints of anxiety, depression, post-traumatic symptoms, eating disorders, etc. A brief description of the transgenerational transmission of trauma is presented.

## 1 Introduction

In every family there are events that affect the fate of the next generations and thus form the attitude to life, to the world and others, to success and failure, predetermine the choice of an occupation, of a partner. The transgenerational approach in psychology and psychotherapy is based on Freud’s ideas that individual human experience bears the imprint of the experience added by previous generations. Collective trauma is associated with a shared psychological and emotional experience that affects a large group of people or an entire community as a result of a devastating event, such as a natural disaster, acts of terrorism, war and genocide, violence, etc. Intergenerational trauma, also known as transgenerational trauma or inherited trauma, refers to the transmission of trauma from one generation to another (Yehuda and Lehrner, 2018).

There are several basic mechanisms for the transmission of trauma across generations. Attachment is assumed to have a mediating role in the transgenerational transmission of abusive and neglectful behaviors. Mothers’ styles of attachment are passed on to their children. Traumas of attachment in childhood, negative experiences that affect one generation, are likely to affect subsequent generations, as well (Gravener et al., 2012; Özcan et al., 2016).

Intergenerational patterns of insecure attachment have also been widely substantiated, particularly when examining the impact of parents’ unresolved trauma on their children. The presence of unresolved trauma or loss can impair the mother’s ability to respond sensitively and effectively to the infant’s needs and increase the risk of developing an insecure attachment. Unresolved trauma or loss can alter a mother’s expectations and perceptions of her child, as well as her ability to respond sensitively, thereby compromising the infant’s development of secure attachment (Iyengar et al., 2014).

According to Payne and Brooks (2019), insecure attachment styles are associated with adverse experiences in childhood that result in medically unexplained symptoms and the ability to self-regulate. The language of trauma, also called “primary language” finds expression not only verbally (Wolynn, 2017). Its manifestation can have different dimensions: physical, physiological, emotional, and behavioral. Physical symptoms are chronic for which no acceptable medical explanation has been found. In the presence of a traumatic event in childhood—protracted or one-time, memory function is hindered. Emotionally saturated information remains unclassified and named through language, undeclared, i.e., implicit (Van der Kolk, 2014). On the other hand, style of attachment is known to be stored in implicit memory.

When there is a real or perceived threat to survival, well-being, or safety, attachment behaviors are engaged in order to reduce distress. This is accomplished by seeking greater proximity to the attachment figure to provide comfort and security (Schachner et al., 2005). Adverse circumstances in early childhood are a powerful risk factor for both poor physical health and psychopathological symptomatology in adulthood (Adshead and Guthrie, 2015). Adults form comfort-seeking and reassurance behaviors by the way of healthy self-care through others. An insecure attachment style was found to be associated with frequent visits to primary care facilities (Taylor et al., 2012).

Medically unexplained symptoms, such as fatigue, chest pain, headache, dizziness, nausea, back pain, insomnia, shortness of breath, abdominal pain and tingling were more often reported by women. They are also found in a population where the language is not native (Steinbrecher et al., 2011). There is a tendency to identify with symptoms, greater social isolation (Dirkzwager and Verhaak, 2007), symptoms of anxiety, depression and somatization (Löwe et al., 2008).

The negative collective experience of an entire community influences the transmission of traumatic experiences to subsequent generations through family secrets, untold stories of loss, murder, torture, etc. During the totalitarian political regime in the period 1984–89, the Bulgarian Turks were in a serious ethnic, cultural and religious crisis. It referred to taking away their native Muslim/Turkish names and renaming them with Christian names, banning them from communicating in their native Turkish language, banning them from observing cultural and ethnic traditions (Bakalova, 2006). As a result of this severe crisis, many Bulgarian Turks left the country. Many untold stories and secrets referred to this period. For this ethnic community, this period represented a serious crisis that had the character of a cumulative trauma and had its consequences in the following generations.

There are several basic mechanisms for the transmission of trauma across generations. Abraham and Torok (2005) describe the mechanism of the so-called “endocrypt identification.” It has been found that people who have experienced trauma often develop anxiety, depression, signs of post-traumatic stress disorder, feelings of threat and unpredictability of the future, and an appreciation of the negative in themselves and in the world. Children of parents with severe trauma demonstrate the same disorders, and their parents have approximately the same parenting styles, significant vulnerability in stressful situations, feelings of guilt, anxiety, depression, phobias and panic reactions. Traumatized parents can directly “stimulate” trauma in their children and they can demonstrate symptoms that are contained in the parents’ traumatic experiences. The unacceptable, unconscious, and unnamed content is transferred into the other’s psyche. This provokes the subject to act as if these fears and fantasies belong to him/her. Lebovici (1993) describes this process and introduces the concept of “family mandate,” which is given to the child at birth and defines his position and role in the family. Stierlin et al. (1979) and Boszormenyi-Nagy (2014) talk about parents’ unconscious assignment to children of roles and tasks related to correcting a mistake, alleviating suffering, mitigating anxiety, etc. This implies loyalty to parents and ancestors, which forms a strong bond between generations, expressed through the so-called “family accounts” where the next generation owes a debt to the previous one. If these “debts” accumulate over generations, each new member of the family is already burdened with a heavy inheritance from birth. Tisseron (1996) describes the influence of the secret on the child’s psyche. All family secrets are perceived by the child as violence that will not be forgotten and that will “weigh” on their whole mental life and will affect their professional, intimate, and social life. Modern views on the transgenerational transmission of trauma are related to the development of J. Bowlby’s theory of attachment. F. Ruppert, combining the theories of trauma and attachment, makes the connection between the transmission of the traumatic experience, which continues to work in subsequent generations. This can appear as a separation of parts of the personality of the survivor self and the wounded self, in which all the information about the trauma is available in the form of serious illnesses, such as important signals and symptoms that cannot be alleviated by medical treatment.

The main differences between symptoms caused by cumulative trauma and those caused by single traumatic events are that the former lead to changes in the regulation of affects, different types of somatization, psychogenic pain symptoms, somatoform and conversion symptoms, depersonalization and derealization, and persistent dissociative disorders of identity, such as amnesia, fragmentation of ego status, and dissociative identity disorder (Van der Kolk et al., 2005). According to Schore (2009), attachment trauma can produce effects similar to those of other forms of developmental trauma. The pathogenetic model based on attachment trauma provides an explanatory model for linking early relational trauma to psychopathological processes of a complex clinical picture. A parent’s neglect, threat, or outright abuse causes the development of dysfunctional beliefs and expectations that are typical symptoms of a traumatic developmental disorder or complex post-traumatic stress disorder (Van der Kolk et al., 2005). Insecure attachment and low mentalizing capacity mediate the relationship between childhood trauma and dissociative experiences (Huang et al., 2020).

## 2 Methods

The presented case makes it possible to trace and analyze the tendency for transgenerational transmission of the attachment model and traumatic experience in three generations in the female line.

The methods used to achieve the goal are:

- *Case description*

A case is presented of a young woman, M., 25 years old, graduate of Master’s degree in psychology, who sought psychological help for panic attacks, claustrophobia, social anxiety and fear of intimacy. She is a representative, third generation, of people affected by the political regime, which is presented in the theoretical part. This part of her personal history is not the purpose of the study, but the mechanisms of transmission of the traumatic experience, and how they relate to her current condition. Her medical history is presented.

Following the signing of informed consent, audio recordings were made of *the questionnaire and the semi-structured interview* of M. with her ancestors, three generations back. These recordings were transcribed and subjected to descriptive and content analysis. As a first step, M. answered the questions included in the two methods used. At the end of each step, she gave feedback on the process.

- *Questionnaire for identification and awareness of significant events and generational traumas* related to loss, rejection, illness, hospitalization, etc. The questionnaire was completed by the main participant in the study, with the help of her ancestors, three generations back. The purpose is to confirm the assumption of cumulative trauma as one of the predictors of mental suffering of M.
- *Semi-structured interview* three generations back in the female line. For the third generation, the Turkish language is used as a channel of communication, after which the narrative is transcribed and translated into the Bulgarian. The interview consists of 30 questions structured in four sets. The emotional experiences of three female generations are examined: *quality of attachment to significant others in childhood; pre- and perinatal emotional status of the mother; emotional status of the mother in the first 3 years of the child’s life; intergenerational trauma*. The purpose is to infer internal working models of attachment, transmitted across generations, as another possible predictor of mental suffering of M.

## 3 Results

### 3.1 Case description

It concerns M., a 25-year-old Bulgarian woman of Turkish origin, recently married, whose husband is also of Turkish origin. M. has a Master’s degree in Psychology. Trained in a cognitive-behavioral psychotherapy paradigm.

She grew up in a full family, Bulgarians of Turkish origin. Has a younger sister. During the political regime, the names of her parents and their parents were changed (the events took place several years before her birth). Her parents’ families discussed leaving the country.

M. separated from her family when she was 19 years old to get her education in Psychology. Her social circle is narrow, she has limited interests outside of her studies.

The first meeting with her coincided with the global health pandemic crisis of Covid19 and her upcoming wedding. In addition to her main complaints related to mixed anxiety disorder, current topics are as follows: non-integrated parental figures, autonomy, self- perception, social identity. Before seeking help, her suffering confined her to the home, and to the illness, making relatives to take care of her. She often cried and withdrew into solitude.

During the study, M. was motivated, cooperative and contributory, with high intellectual and cognitive potential.

Medical History: M. was born with blue asphyxia. The development of general motor skills corresponds to age norms. She started speaking in Turkish, but gradually mastered both languages. At the age of two, she was hospitalized due to severe otitis media. At the age of 10, she was hospitalized again due to impaired glucose tolerance and neurogenic bladder. At the age of 13, there were persistent migraine complaints, palpitations, shortness of breath, “pain in the heart” and chest area, at the age of 14—suspected asthma. Shortness of breath was especially strong in rainy weather, there was often a slight cough when moving and lying down, which gave rise to a new hospitalization due to suspicions of bronchitis. During inhalation she suffered a severe attack of shortness of breath and subsequent dissociation. At the age of 15, she was hospitalized in child psychiatry and diagnosed with mixed dissociative (conversion) disorder.

During all her hospitalizations, her mother was by her side.

### 3.2 Questionnaire for identification and awareness of significant events and trauma in the generations

Data in the questionnaire points to traumatic events that took place in past generations (vulnerability in relation to the preservation of the native language, identity, choice of intimate partners of the same ethnicity, punishments accompanied by anger, fear and somatic reaction by the interviewees). Traumatic events can be traced both in the female and male lines—traumatic losses that remained unrepentant, a ban on observing religious rituals, mental disorders, termination of pregnancy in the third generation back. M. was tolerated by her grandmother as her own daughter, which violated family boundaries. In the female line, traumas were related to both biological and social factors—termination of pregnancy, losses at an early age that were not talked about in generations, parentification—siblings took care of siblings, violence, disregard of the child’s wishes. There were somatic and mental diseases that blocked the possibility of accessibility and responsiveness of the attachment figure—“hypochondria,” escape into illness, heightened blood pressure, disc herniation, oncological diseases. The combination of biological and social factors resulted in the male line in problems with the identity, the role in the family, which blocked the possibility of providing support for the attachment figure.

### 3.3 Interview about emotional experiences

The data in the interview in the three generations of female figures shows emotional encapsulation, high levels of anxiety, lack of responsiveness to the needs of the child, and rebellion against one’s own position in the family, which is rationalized and explained by religious affiliation.

#### 3.3.1 Quality of attachment with significant others in childhood

The predominant emotions of all three generations of female figures were of sadness and anger, unrecognized by the attachment figure and unreacted. As children, members of this lineage, they were assigned age-inappropriate responsibilities. The attachment figure’s lack of tenderness and responsiveness to children’s needs is clearly stated. Childhood memories were pushed out. The child was anxious, suffering, lost, with unsatisfied and unrecognized emotional and physiological needs.

#### 3.3.2 Pre- and perinatal emotional status of the mother

Imputed responsibilities continue in this period as well. There is a lack of emotional connection between the mother-to-be and her future baby. There was also a lack of emotional support from future fathers in previous generations. The sacral moment of birth and entry of the new member into the family was neglected. Women were quickly returning to field work.

#### 3.3.3 Emotional status of the mother in the first 3 years of the child’s life

Emotional needs have found expression in somatic symptoms. In the third generation, there was a somatic disease related to heart activity, in the second generation, anxiety to the point of fear of hospitals and doctors was registered. (With M., frequent hospitalizations are a fact). In the second generation, there was also fear of abandonment by the family due to political events (many families left the country as a result of political events).

#### 3.3.4 Intergenerational trauma

In the first generation in the female line, high control and inconsistency with one’s own desires and needs clearly stood out. Control by male figures was accepted conciliatoryly, but was demonstrated as controlling the child and disregarding its needs. The image of the male figures was in an ambivalent position, accepting and understanding and at the same time imposing and rejecting. In the second generation, the male figures possessed an antisocial personality structure, depressive symptoms, and deviant behavior. In this dynamic, in a family context, the child was suffering, rejected, misunderstood, which resulted in somatic problems, and in the third generation—in psychopathology, with derealization and depersonalization. The attachment figure in the third generation was in an ambivalent position, recognizing the illness but relying on external factors to overcome it. The attitude toward the father figure in the third generation was ambivalent. The father was understanding but lacked support skills, which caused anger.

## 4 Discussion

Because of inconsistent and inadequate responses to the needs of the young child, the latter does not develop healthy, positive, and functional internal working models, including a set of beliefs or expectations about himself and others that govern affect, cognitions, and interpersonal behavior.

### 4.1 Trauma transmission mechanisms—through abuse and neglect, forming a shaky internal working model

Attachment trauma in all three generations of female figures has a negative impact on internal working models and the formation of a negative self-image. There is a negative and/or incoherent sense of self, they feel worthless, inadequate, with low emotional differentiation. The desires listed in the narrative express needs to connect and to receive love and care that were absent in earlier stages of life.

The anxiety experienced by the child in the context of early caregiving activates behaviors through which he seeks closeness to the caregiver. If these behaviors remain neglected, the child evaluates the caregiver as emotionally unavailable and rejecting and develops coping strategies, such as avoidance, and builds for himself an internal working model of undeserving love and care. Internal working models reflect the history and experiences a person has in their relationship with the primary caregiver during childhood. This makes them very resilient and impactful on relationships later on. In adulthood, they also serve as a script or scenario that guides how to behave in social situations, in intimate and sexual relationships.

In traumatic events or insensitive, pathogenic caregiving, especially when associated with abuse or neglect, the infant cannot rely on a trusted caregiver to turn to in perceived danger, blocking the bonding process. Misinterpretations, unavailability or unresponsiveness of the mother prevents the baby from gaining experience of self-monitoring and communication, which results most often in an insecure type of attachment or attachment disorders and leads in adulthood to personality disorders and interpersonal dysfunctions.

### 4.2 Manifestations of trauma through bodily symptoms concerning physical health

Parenting forming insecure attachment has various dimensions and manifestations and is tied to a transgenerational history of inadequate caregiving. In this case, unsatisfactory parenting is linked to trauma for the child—mental, emotional and neglect. This results in fear, helplessness, dissociation, confusion—experiences that are intense enough to have a long- term negative effect on attitudes, emotions and behavior. It is attachment that best describes the interaction between development, trauma, and dissociation. Relational trauma at an early age triggers states of fearful hyperarousal and dissociative defense mechanisms. When the care system is unable to buffer the experiences of these children it becomes a source of distress. Trauma triggers archaic behavioral systems to protect the individual in life- threatening situations. When the archaic defense system reaches the extreme escape response, it causes a separation of conscious experience from the usual sense of self—these are the so-called dissociative symptoms of depersonalization and derealization, described as dissociative detachment.

In the described case, the history of the first generation had a chronic illness defined as heart failure, which formed a pattern of manipulative behavior toward the second generation. This implies a reversal of parent–child roles, where the child is required to provide comfort to the primary caregiver. M., who is the third generation, has many somatic complaints, for which she has been hospitalized many times. Symptoms “migrate” throughout the body so that the body serves the mental discomfort. It is an endless scenario to repair the emotional pain and neglect in the first generation through the female line. This line of invisible and unconscious family loyalty could answer M.’s question about the need to seek attention through illness accompanied by anger and dissatisfaction. When the traumas are over a period of time and tend to recur, then the child is in a state of anxious anticipation of painful experiences and gradually accepts the fact that the primary caregiver is both a source of threat and protection. The relationship of affective, cognitive, physiological, and behavioral components of attachment trauma transmitted across generations is clearly evident.

*Transgenerational trauma in the context of cumulative trauma* experienced by the study participant (third generation) as a result of the political regime is not a leading predisposition for the suffering of M. But, it certainly brings the shadow of the unspoken, the unexperienced. She finds a response in a social context—in her childhood, M. had an experience of a game based on ethnic division, which also affected her social identity in early adulthood. The children, who are the third generation of both the victim and the other side, turn children’s play into a reenactment of the events of their ancestors: some set standards, others obey. In the described case, the traumatic experience passed down through the generations has the character of loyalty to the ancestors, as well.

Transgenerational traumas are passed from generation to generation only through the process of attachment formation. Failure to recognize and respond to children’s needs results in insecure attachment and the development of dysfunctional internal working models. Sensitivity implies “reliance” on the infant’s cues, accessibility and reactivity to those cues, especially in situations requiring regulation of the infant’s anxiety, arousal, affect, and stress. A state of detachment caused by trauma can prevent integration of the traumatic event and cause long- term fragmentation in consciousness, memory, identity, and body image—symptoms described as dissociative compartmentalization.

In the end of the study M. shares: “I learned to BREATHE—freely and stand up for myself. I saw how brave I am, how I can rebel against authority figures and defend myself without being worried. I heard my pleasant voice—I began to enjoy it. I traveled to Turkey by bus (I wasn’t alone), but I made it. I smelled the grass after the rain, danced in the rain at my cousin’s wedding and enjoyed every minute of it. I realized that rain is not the worst thing in the world. I could eat delicious food and enjoy it, as well as my stomach and my soul. I realized that the stomach and all the pains appeared because I avoided hearing the voice of my soul, hearing its needs and desires, and perhaps most importantly, which even surprised me what a manipulator I can be—to “punish others for what they have done to me” and to receive the necessary attention from them.”

The process of disidentification from the traumatic experience of the ancestors allows to recover the history belonging to the past. This is the way for the subject to gain freedom in the formation of his identity and individuality. Incorporating ancestral trauma into one’s subjective world allows traumatic experiences to be integrated and transformed into structuring rather than destructive ones.

## Data availability statement

The raw data supporting the conclusions of this article will be made available by the authors, without undue reservation.

## Ethics statement

Written informed consent was obtained from the individual(s) for the publication of any potentially identifiable images or data included in this article.

## Author contributions

ZK: Writing – review & editing, Writing – original draft. VM: Writing – review & editing, Writing – original draft.
